# Monomeric Bistability and the Role of Autoloops in Gene Regulation

**DOI:** 10.1371/journal.pone.0005399

**Published:** 2009-04-30

**Authors:** Stefanie Widder, Javier Macía, Ricard Solé

**Affiliations:** 1 Complex Systems Lab (ICREA-UPF), Barcelona Biomedical Research Park (PRBB-GRIB), Barcelona, Spain; 2 Santa Fe Institute, Santa Fe, New Mexico, United States of America; Center for Genomic Regulation, Spain

## Abstract

Genetic toggle switches are widespread in gene regulatory networks (GRN). Bistability, namely the ability to choose among two different stable states, is an essential feature of switching and memory devices. Cells have many regulatory circuits able to provide bistability that endow a cell with efficient and reliable switching between different physiological modes of operation. It is often assumed that negative feedbacks with cooperative binding (i.e. the formation of dimers or multimers) are a prerequisite for bistability. Here we analyze the relation between bistability in GRN under monomeric regulation and the role of autoloops under a deterministic setting. Using a simple geometric argument, we show analytically that bistability can also emerge without multimeric regulation, provided that at least one regulatory autoloop is present.

## Introduction

Bistability is known to pervade key relevant biological phenomena [1]. Many relevant examples can be found including, e.g. the determination of cell fate in multicellular organisms. This occurs with *Xenopus* oocytes, which convert a continuously variable concentration of the maturation-inducing hormone progesterone, into an all-or-none biological maturation response [2]. Stem cells on the other hand present a switch where the expressions of the involved transcription factors (OCT4, SOX2, and NANOG) are stabilized by a bistable switch. When they are expressed and the switch is on, the self-renewal genes are on and the differentiation genes are off. The opposite holds when the switch is off [3]. A third example is the cell-cycle regulation, which exhibits a temporally abrupt response of Cdc2 to non-degradable cyclin B [4]. This capacity of achieving multiple internal states is at the core of a plethora of regulatory mechanisms, often associated to small genetic circuits, including both switches [5], [6], [7], [8], [9] and oscillators [10], [11]. Understanding their logic and how it changes under parameter tuning are two important goals of systems biology.

**Figure 1:**
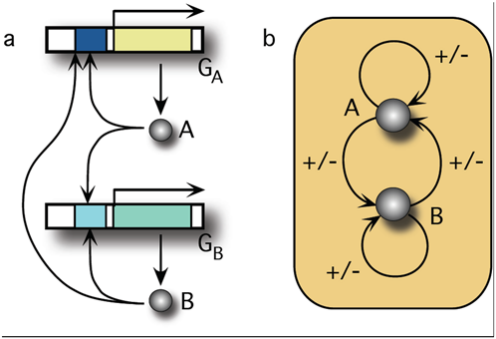
Schematic representation of a general genetic circuit with two components. In (a) a genetic circuit with monomeric autoloops and cross-regulation involving two genes (G_A_, G_B_) coding for two proteins (A, B) acting as transcription factors. Under certain conditions, this type of genetic circuit can show bistability. Here all possible regulatory modes are shown (+/−). (b) Simplified diagram summarizing the logic of this system.

A general consensus indicates that such switches are based on a mutual regulation of two transcription factors (figure 1), e.g. mutual inhibition: protein A inhibits the synthesis of protein B and vice versa [12]. Depending on the type of regulation they can be in two different stable states and may change from one to the other spontaneously or due to an external signal [12], [13], [14], [15]. For example, during the embryonic development of *Drosophila melanogaster* the expression of the *hp* gene responsible for *hunchback* formation is activated by Bicoid (Bcd) protein. In early embryogenesis, the diffusion of Bcd, translated from the mRNA located at the anterior end of the egg, forms an exponential concentration gradient, establishing the anterior–posterior axis. Upon this signal, a bistable mechanism allows for large changes in *hb* promoter occupancy under small changes in Bcd concentration across some threshold generating an on–off expression pattern. This bistable mechanism explains the sharpness of the Hb expression, from highest to lowest values taking place in a spatial scale spanning just 10% of the egg length [16]. In other natural scenarios bistability can be implemented by non-transcription factors. However, even in these cases mutual regulation is required. An example of bistable systems based on mutual inhibition of non-transcription factors can be found in signalling pathways. In *Saccharomyces cerevisiae*, signal transduction pathways involved in sensing external stimuli often share the same or homologous proteins, e.g. high osmolarity pathway and pheromone pathway. Despite potential cross-wiring, cells show specificity of response. This specificity can be achieved, among other mechanisms, by mutual inhibition of the shared proteins. When a single cell is exposed to osmostress and pheromone induction simultaneously, only one of the two pathways is activated inhibiting the activation of the other pathway. In this case, the activated pathway corresponds to one of the two possible stable states of the bistable system (see [17] and references therein).

Focusing on two-components genetic circuits, their regulatory proteins are known to form homodimers (or multimers) to be effective transcription factors allowing to turn ON or OFF the state of target genes [12], [18], [19]. Multiple examples can be found in natural systems e.g. in the lambda-phage where the change from lysogenic to lytic behaviour in response to environmental changes is regulated by a switching two-component circuit. In this case, the two transcription factors involved, CI and Cro, must form homodimers to be effective [20], [21]. The same requirements allow for bistability in synthetic designs. This is the case of the genetic toggle switch in *Escherichia coli*, where bistability of the toggle arises from the mutually inhibitory arrangement of the repressor genes. The regulatory transcription factors TetR and LacI form homodimers and homotetramers respectively. The transition from one to the other stable state is triggered by external inducers (aTc and IPTG) [6].

For general systems without any specific assumptions, multimeric regulation was assumed to be essential to obtain bistable behaviour [22], [23]. The inability to exhibit bistability in monomeric circuits without autoloops, was previously demonstrated [24]. These results indicate that linear or Michaelis-Menten kinetics cannot provide bistability and higher degree of non-linear genetic regulation is required. Different mechanisms can introduce this non-linearity. Positive cooperativity of binding is one such mechanism. It can result from non-independent binding at two adjacent operator sites. A similar effect results if a repressor is effective only as a dimer (or multimer) and the monomer-monomer affinity is weak [24]. Several models of bistable systems involving only positive regulation also require cooperativity of binding [25], [26]. Despite the above, monomeric bistability has been found in particular, bimolecular systems with Michaelis-Menten kinetics under the indispensable key-assumption of constancy of the total amount of proteins [27]. Also, some kind of multistability is possible in a stochastic scenario without cooperative binding [13], [28], but under fully symmetric interactions. However, the flips between the two states are also stochastic and the observed alternative states cannot be stabilized (as it occurs in real biological switches) due to the effectively monostable character of the system without noise.

In deterministic dynamics, bistability requires the existence of three fixed points. In this paper we demonstrate, to our knowledge for the first time, that deterministic bistability can emerge for two-component gene circuits by considering solely auto-regulatory loops. This is unlike the previously briefly mentioned cases [13], [27], where bistability is not generated by the intrinsic topology of the circuits. In other words, we demonstrate that bistability in monomeric two-component circuits can be implemented exclusively by the topology of interactions, with no additional constraints, being a single autoloop enough to obtain it. Our analysis is based on simple geometrical features associated to the system's nullclines and their crossings. As shown below, the presence of an autoloop introduces essential geometrical constrains responsible for the existence of three fixed points. Our results can help understanding the essential role of autoloops in small natural circuits and their synthetic counterparts.

## Results

### Geometrical features

**Figure 2:**
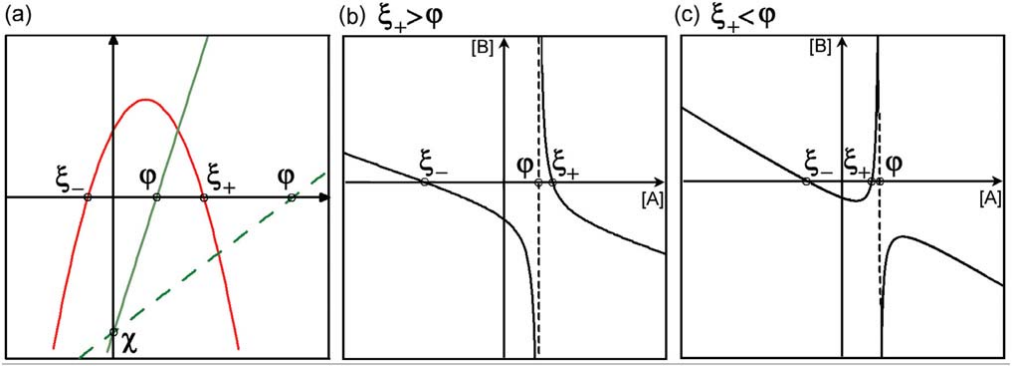
Qualitative shapes of the nullclines. (a) Graphical representation of the nullcline's components. The numerator is the parabolic curve and the denominator the straight line. Two feasible scenarios are shown: the solid line denotes ξ^+^>ϕ, the dashed line corresponds to ξ_+_<ϕ. (b),(c) Qualitative behaviour of the nullclines applying the two possible conditions.

In order to perform a general analysis of the nullclines, as introduced in Materials and Methods, we study the single components (numerator and denominator) of the expressions independently, see figure 2(a). The numerator is a parabolic function having two analytically well defined crossing points (ξ_+_>0 and ξ_−_<0 ) with the horizontal axis given by
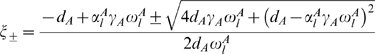
(4)


The denominator is a lineal function crossing the horizontal axis in ϕ = γ_A_α^B^_c_/d_A_. The points ϕ and ξ_+_ are the upper and lower bound of the protein concentrations of the system within the biological meaningful region. Combining the two components, two different scenarios are feasible, ϕ<ξ_+_ or ϕ>ξ_+_, comprising different geometrical features. In both cases we find two crossing points with the horizontal axis in ξ_±_, no inflection points, and the nullclines tending towards their oblique asymptotes with an identical slope *m* = −ω^A^_l_/ω^B^_c_ for both settings A→±∞. From this expression we see that the autoloop is related with certain geometrical features. Systems without auto-regulatory loops (ω^A^_l_ = 0) do not exhibit oblique asymptotes, but horizontal. As shown later the existence of oblique asymptotes is closely related with the number of possible fixed points and bistability.

In the first case, ξ_+_>ϕ, we obtain a vertical asymptote in ϕ with its lateral behaviour given by lim_A→ϕ±_(B)_dA/dt = 0_ = ±∞ For the second case, ξ_+_<ϕ, we find similar asymptotes with opposite lateral behaviour according to lim_A→ϕ±_(B)_dA/dt = 0_ = ∓∞. In order to determine possible extrema of the nullcline (dB/dA = 0), we find, after some algebra, that the inequality
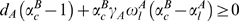
(5)must be met to provide valid solutions, hence extrema. Rewriting the conditions ϕ>ξ_+_ and ϕ<ξ_+_ by using the previous expressions for ϕ and ξ_+_ we conclude that only ϕ>ξ_+_ satisfies condition (5) and hence provides extrema. However, according with the vertical asymptotic behaviour and the existence of only one crossing point (ξ_+_) within the positive domain, we conclude that the extrema are located within B<0. Hence, no extrema can be obtained within the biologically meaningful domains, i.e. by imposing the biological constraint that the levels of proteins must be positive (A>0, B>0), for either scenario. In figure 2(b),(c) the two different types of possible behaviour are shown. Furthermore, a similar analysis has been performed for a system without basal transcription and the geometrical features are not affected qualitatively.

### Fixed point analysis

**Figure 3:**
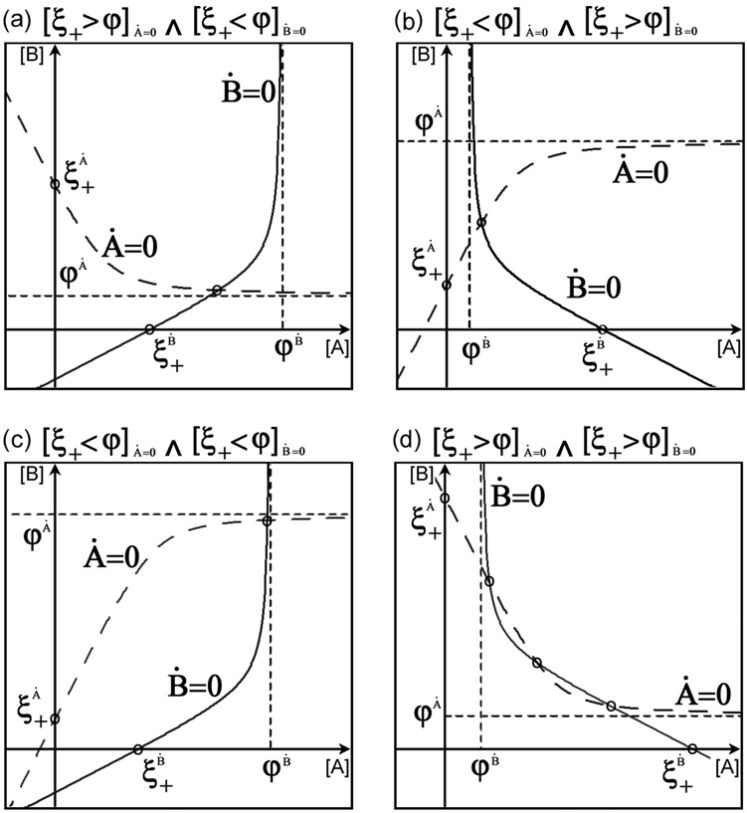
The four possible scenarios of nullcline combinations. Dashed line corresponds to nullcline dA/dt = 0, solid line to dB/dt = 0, ϕ^A^ and ϕ^B^ denote the location of the asymptote for dA/dt = 0 and dB/dt = 0, respectively. Due to the symmetry of the nullclines' expressions, the vertical asymptote of dB/dt = 0 corresponds to the horizontal of dA/dt = 0. Analogously, ξ^A^_+_ and ξ^B^_+_ are the crossing points with the axis. (d) The geometrical features of the nullclines allow for two possible cases. Three crossing points (depicted) or a single crossing (not depicted).

Using the previous geometrical approach, we are in the position to reassemble both nullclines within the biological meaningful region determining how many crossing points between both nullclines can arise under different regulatory conditions. The crossings between nullclines define the so called fixed points, i.e. the levels of proteins A and B such that dA/dt = 0 and dB/dt = 0 simultaneously, thus no changes in protein concentration will take place Four possible cases are obtained based on the symmetry of the expressions for nullcline dA/dt = 0 and dB/dt = 0. They are shown in figure 3. For the cases [ξ_+_>ϕ]_dA/dt = 0_ ∧ [ξ_+_<ϕ]_dB/dt = 0_ and [ξ_+_<ϕ]_dA/dt = 0_ ∧ [ξ_+_>ϕ]_dB/dt = 0_ (3(a) and 3(b), respectively), equal geometrical arguments apply. In both cases the nullclines exhibit opposite monotonies and opposite curvatures within the entire domain due to the absence of extrema and inflexion points. These conditions solely allow for a single crossing, hence monostability. In the case [ξ_+_<ϕ]_dA/dt = 0_ ∧ [ξ_+_<ϕ]_dB/dt = 0_, depicted in figure 3(c), the nullclines exhibit opposite curvature, but equal monotonies. Again, the absence of extrema and inflection points does not allow for three crossings, however under the special condition of [ξ_+_]_dA/dt = 0_ = [ξ_+_]_dB/dt = 0_ = 0 two crossing point arise. In accordance with expression (4), these conditions can be satisfied, if 4d*_i_*γ*_i_*ω*^i^_l_* = 0 with *i* = {A, B}. Since γ*_i_*>0 and d*_i_*>0, only ω*^i^_l_* can be zero and in this case (for a system without autoloop regulation) the nullclines' expressions now read:
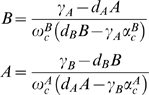
(6)where the fixed points can be analytically solved. The solutions are determined by the roots of a polynomial of second degree allowing for two possible fixed points at most. However, the polynomial crosses the vertical axis at −γ*_A_* γ*_B_*ω*^A^_c_*α*^A^_c_* forcing one of the roots to be located within the negative domain. Hence, without autoloops only monostability is possible in monomeric gene circuits. This result is consistent with analysis previously reported [24]. For the setting [ξ_+_>ϕ]_dA/dt = 0_ ∧ [ξ_+_>ϕ]_dB/dt = 0_ both nullclines show the same type of curvature and monotony. Due to the oblique asymptote, introduced by the autoloop, no analytical constraints prevent the existence of three crossing points. In figure 3(d) we show an example of bistability with monomeric regulation.

**Figure 4:**
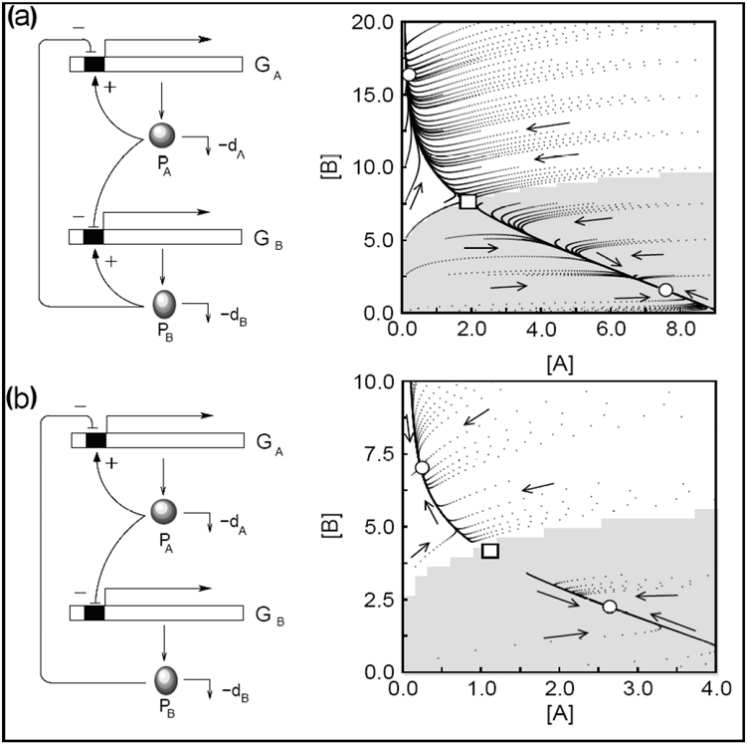
Numerical simulations and stability analysis. In (a) circuit with two autoloops and in (b) circuit with one autoloop are shown. Circle denotes a stable, square an unstable fixed point. The basins of attraction are shown in grey and white. The following sets of parameters have been used: (a) γ_A_ = 1, d_A_ = 1, α^A^_l_ = 10, ω_l_^A^ = 1, ω^B^_c_ = 1, α^B^_c_ = 0, γ_B_ = 1.1, d_B_ = 0.1, α^B^l = 2.1, ω^B^_l_ = 0.1, ω^A^_c_ = 1.1, α^A^_c_ = 0 and (b) γ_A_ = 5, d_A_ = 8, α^A^_l_ = 9, ω_l_^A^ = 1, ω^B^_c_ = 1, α^B^_c_ = 0, γ_B_ = 8.5, d_B_ = 1, α^B^_l_ = 0, ω^B^_l_ = 0, ω_c_^A^ = 1, α^A^_c_ = 0.

In order to determine the impact of the number of autoloops on bistability, we have numerically analyzed the effect of downsizing the system from two to one autoloop (ω*^i^_l_* = 0, ω*^j^_l_* = 0). As figure 4 shows, only one autoloop is required to allow bistability. In figure 4(a) the nullclines of a circuit with two autoloops are depicted and three fixed points appear for a given set of parameters. The stability analysis reveals two stable fixed points separated by an unstable one resulting in the corresponding basins of attraction. Figure 4(b) shows a system with a single autoloop. These numerical examples demonstrate that genetic circuits with monomeric regulation are able to exhibit deterministic bistability, whereby only a single autoloop is required to satisfy the necessary geometrical constraints.

### Impact of regulation type on monomeric bistability

In the previous sections the type of regulatory interactions, given by α^i^_l_ and α^i^_c_ was handled generally. However, the individual regulatory interactions, i.e. activation or inhibition, introduce additional constraints for the emergence of bistability. Applying some algebra to condition ϕ<ξ_+_ (bistability), we obtain an equivalent expression as in (5) with the opposite inequality. Focusing on the type of regulation, it can be rewritten as
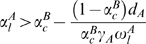
(7)


**Figure 5:**
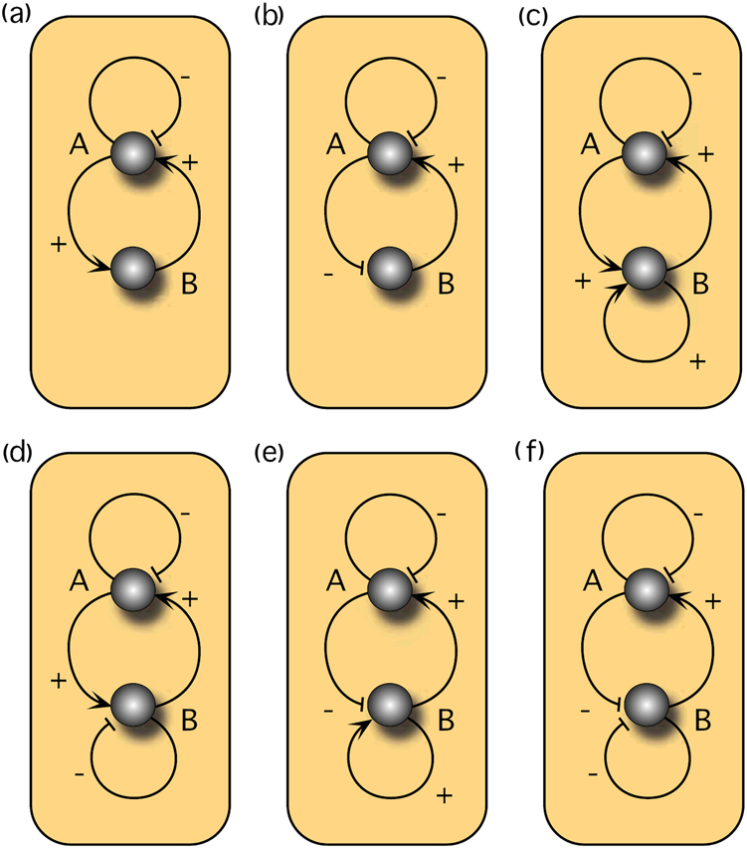
All possible regulatory combinations preventing bistability. In other circuit topologies the emergence of bistability is possible but conditioned to the specific parameters of the system.

This leads us to two different instances: (a) if α^B^_c_>1, then α^A^_l_>α^B^_c_ and (b) if α^B^_c_<1, then α^A^_l_>α^B^_c_ ∨ α^A^_l_<α^B^_c_. As a consequence systems with inhibitory regulation in the autoloop and activatory cross-regulation can not exhibit bistability. In all the other cases no geometric impediments are present. Figure 5 shows all possible regulatory topologies which cannot exhibit bistability, irrespective of the specific set of parameters used.

## Discussion

To summarize, a general, analytic set of conditions for bistability in simple two-element genetic circuits has been derived for monomeric regulation. Although previous work suggested that such kind of mechanism would be unlikely to be observed, here a simple geometric argument reveals that wide parameter spaces allow monomeric regulation to generate multiple stable states. These results permit to predict the expected scenarios where a reliable switch could be obtained. Current efforts in engineering cellular systems [29], [30], [31] would benefit from our general analysis. In this context, although dimerization seems to be a widespread mechanism in GRNs, our study indicates that potential scenarios for monomeric regulation could be easily achieved. The current state of the art in synthetic biology allows for a customized engineering of monomeric transcription factors e.g. Zinc finger TFs can be easily designed to bind different DNA sequences [32]. Building these monomeric transcription factors in a properly designed network [33], the experimental implementation of monomeric bistable circuits seems thus to be feasible.

Finally, further work should explore how noise can act on these types of dynamical systems. In eucaryotic cells, dimerization has been shown to provide a source of noise reduction at least at the level of simple GRNs [34]. Future studies should see how our monomeric circuits are affected by noise and what types of limitations and advantages can be obtained.

## Materials and Methods

### Genetic circuit

We focus our analysis on the most general system formed by two genes. Gene A is expressed under the constrains of two different monomeric regulatory modes. Protein A exhibits an auto-regulatory loop by binding to its own promoter, as well as a cross-regulation mediated by protein B. Gene B expression is analogously regulated (see figure 1). We consider the general case without any specific assumptions about the type of regulatory interactions, i.e. activation or inhibition, but introduce them as a tunable parameters α. The basic dynamical properties of the circuit can be described by the following set of ODEs obtained from the set of biochemical reactions:
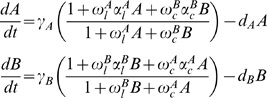
(1)


We are assuming basal transcription, the standard rapid equilibrium approximations supposing that binding and unbinding processes are faster than synthesis and degradation, and constancy of the total number of promoter sites. Furthermore, the concentration of the other biochemical elements involved remains constant during time and can be subsumed in the kinetic constant γ_i_. The binding equilibrium of the autoloop and the cross-regulators are denoted by ω^i^_l_ and ω^i^_c_, respectively. Furthermore α ^i^_l_ and α ^i^_c_ denote the regulatory rates with respect to the basal transcription, for the autoloop and cross-regulation respectively. Values<1 correspond to inhibitory regulation, whereas >1 accounts for activation. Finally, d*_i_* is the degradation rate of protein *i*. For a detailed description of this type of calculus, see [21].

### Nullcline analysis

In order to analyze the system's dynamics we obtain the following expressions for the nullclines imposing dA/dt = 0 and dB/dt = 0 considering monomeric regulation:
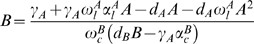
(2)

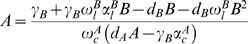
(3)


The number of crossing points between (2) and (3) defines the number of different fixed points within the system. Both nullclines have mathematically symmetric expressions, tunable by the set of parameters. This symmetry facilitates their analysis due to interchangeability of the characteristic features. Hence, the problem can be evaluated by reducing the analysis to one expression. Here (2) is analyzed.

## References

[1] Laurent M, Kellershohn N (1999). Multistability: a major means of differentiation and evolution in biological systems.. Trends Biochem Sci.

[2] Ferrell JE, Machleder EM (1998). The biochemical basis of an all-or-none cell fate switch in Xenopus oocytes.. Science.

[3] Chickarmane V, Troein C, Nuber UA, Sauro HM, Peterson C (2006). Transcriptional dynamics of the embryonic stem cell switch.. PLOS Comput Biol.

[4] Pomerening JR, Sontag ED, Ferrell JE (2003). Building a cell cycle oscillator: hysteresis and bistability in the activation of Cdc2.. Nature Cell Biol.

[5] Dubnau D, Losick R (2006). Bistability in bacteria.. Molecular Microbiology.

[6] Oppenheim AB, Kobiler O, Stavans J, Court DL, Adhya S (2005). Switches in bacteriophage lambda development.. Annu Rev Genet.

[7] Tian T, Burrage K (2004). Bistability and switching in the lysis/lysogeny genetic regulatory network of bacteriophage lambda.. J Theor Biol.

[8] Becskei A, Seraphin B, Serrano L (2001). Positive feedback in eukaryotic gene networks: cell differentiation by graded to binary response conversion.. EMBO J.

[9] Hasty J, McMillen D, Collins JJ (2002). Engineered gene circuits.. Nature.

[10] Elowitz MB, Leibler S (2000). A synthetic oscillatory network of transcriptional regulators.. Nature.

[11] Hasty J, Dolnik M, Rottschäfer V, Collins JJ (2002). Synthetic gene network for entraining and amplifying cellular oscillations.. Phys Rev Lett.

[12] Gardner TS, Cantor CR, Collins JJ (2000). Construction of a genetic toggle switch in Escherichia coli.. Nature.

[13] Lipshtat A, Loinger A, Balaban NQ, Biham O (2006). Genetic toggle switch without cooperative binding.. Phys Rev Lett.

[14] Tian T, Burrage K (2006). Stochastic models for regulatory networks of the genetic toggle switch.. Proc Natl Acad Sci USA.

[15] Santillán M, Mackey MC (2004). Why the lysogenic state of phage lambda is so stable: a mathematical modeling approach.. Biophys J.

[16] Lopes FJP, Vieira FMC, Holloway DM, Bisch PM, Spirov AV (2008). Spatial Bistability Generates *hunchback* Expression Sharpness in the *Drosophila* Embryo.. PLoS Comput Biol.

[17] McClean MN, Mody A, Broach JR, Ramanathan S (2007). Cross-talk and decision making in MAP kinase pathways .. Nat Genet.

[18] Warren PB, ten Wolde PR (2004). Enhancement of the stability of genetic switches by overlapping upstream regulatory domains.. Phys Rev Lett.

[19] Widder S, Schicho P, Schuster P (2007). Dynamic patterns of gene regulation I: simple two-gene systems.. J Theor Biol.

[20] Hasty J, Pradlines J, Dolnik M, Collins JJ (2000). Noise-based switches and amplifiers for gene expression.. Proc Natl Acad Sci USA.

[21] Hasty J, Isaacs F, Dolnik M, McMillen D, Collins JJ (2001). Designer gene networks: Towards fundamental cellular control.. Chaos.

[22] Warren PB, ten Wolde PR (2005). Chemical models of genetic toggle switches.. J Phys Chem B.

[23] Walczak AM, Sasai M, Wolynes PG (2005). Self-consistent proteomic field theory of stochastic gene switches.. Biophys J.

[24] Cheery JL, Adler FR (2000). How to make a Biological Switch.. J Theot Biol.

[25] Tyson JJ, Othmer HG, Rosen R, Snell FM (1978). The dynamics of feedback control circuits in biochemical pathways.. Progress in theoretical Biology.

[26] Wolf DM, Eeckman FH (1998). On the relationship between genomic regulatory element organization and gene regulatory dynamics.. J theor Biol.

[27] Tyson JJ, Chen KC, Novak B (2003). Sniffers, buzzers, toggles and blinkers: dynamics of regulatory and signaling pathways in the cell.. Curr Opin Cell Biol.

[28] Loinger A, Lipshtat A, Balaban NQ, Biham O (2007). Stochastic simulations of the repressilator circuit..

[29] Ferrell JE (2002). Self-perpetuating states in signal transduction: positive feedback, double-negative feedback and bistability.. Curr Opin Chem Biol.

[30] McDaniel R, Weiss R (2005). Advances in synthetic biology: on the path from prototypes to applications.. Curr Opin Biotech.

[31] Solé R, Munteanu A, Rodriguez-Caso C, Macia J (2007). Synthetic protocell biology: from reproduction to computation.. Phil Trans R Soc London B.

[32] Isalan M, Klug A, Choo Y (2001). A rapid, generally applicable method to engineer zinc fingers illustrated by targeting the HIV-1 promoter.. Nat Biotechnol.

[33] Noireaux V, Bar-Ziv R, Libchaber A (2003). Principles of cell-free genetic circuit assembly.. Proc Nat Acad Sci USA.

[34] Bundschuh R, Hayot F, Jayaprakash C (2003). The role of dimerization in noise reduction of simple genetic networks.. J Theor Biol.

